# Preparation of serum capped silver nanoparticles for selective killing of microbial cells sparing host cells

**DOI:** 10.1038/s41598-021-91031-7

**Published:** 2021-06-02

**Authors:** Rehana Parveen, Prasanta Kumar Maiti, Nabendu Murmu, Alokmay Datta

**Affiliations:** 1 Department of Microbiology, Institute of Post-Graduate Medical Education and Research, Calcutta, 700020 India; 2 Department of Signal Transduction and Biogenic Amines, Chittaranjan National Cancer Institute, Calcutta, 700026 India; 3 Advanced Mechanical and Materials Characterization Division, CSIR-Central Glass and Ceramic Research Institute, Calcutta, 700 032 India

**Keywords:** Nanobiotechnology, Medical research

## Abstract

Following access into the cell, colloidal silver nanoparticles exhibit generalized cytotoxic properties, thus appear as omnipotent microbicidal, but not suitable for systemic use unless are free of toxic effects on host cells. The AgNP-Serum-18 when prepared from silver nitrate, using dextrose as reducing and group-matched homologous serum as a stabilizing agent, selective endocytosis, and oxidative stress-dependent bio-functional damages to the host are mostly eliminated. For their bio-mimicking outer coat, there is the least possibility of internalization into host cells or liberation of excess oxidants in circulation following interaction with erythrocytes or vascular endothelial cells. The presence of infection-specific antibodies in the serum can make such nano-conjugates more selective. A potent antimicrobial action and a wide margin of safety for mammalian cells in comparison with very similar PVA-capped silver nanoparticles have been demonstrated by the in-vitro challenge of such nanoparticles on different microbes, human liver cell-line, and in-vivo study on mice model. This may open up wide-range therapeutic prospects of colloidal nanoparticles.

## Introduction

Due to the rising trend of infections with “superbugs”, where no antibiotics have left an option for effective management^1^, suitable nonconventional antimicrobial agents need to be developed. From the beginning of the penicillin era, bacteria have started developing resistance against all newly introduced antibiotics within a few decades. Bacteria accomplish this by a few simple genetic or structural alterations under selective pressure at the target level where the drug could arrest cell replication. So, instead of developing few more selective anti-replicant drug to combat superbugs, a group of resistance-proof, multi-targeted, potent, wide-range cytotoxic agents can be a permanent solution of the issue, provided they spare entry into host cells during systemic use.

By in-vitro studies, almost all heavy metal-based antimicrobial nanoparticles (NP) have shown dose-dependent pan-microbicidal actions against all tested bacteria^2^ fungi^3^, viruses^4^ or parasites^5^ depending on extent of intracellular access. Their unique mechanisms of internalization into microbial and mammalian cells lead to disruption of cellular functions by reacting on different biomolecules at unusual reduced atomic materialistic state^6,7^. These have made them near-ideal new-age antimicrobial agents. However, in the question of the margin of safety, none are suitable for systemic use or environment friendly. For the same reason, their uses remain restricted to some topical applicable drugs for wound management^8^, biofilm-proof indwelling device coating or use as food preservatives, antiseptics and pesticide agents.

An attempt has been made to prepare a systemic usable silver nanoparticle (AgNPs) containing a robust core of aggregated reduced silver atoms (Ag^0^), stabilized by homologous serum components. In circulation, the host’s serum can impart further stability and may not allow release of toxic core materials before internalization into target cells. This is like “targeted nano-missile with a pay-load cluster of atoms”. Due to high zeta-potential, these will be more attracted towards rapidly multiplying, negatively charged bacteria, regenerating cells or cancer cells and will be internalized by forced splitting of the bacterial bi-layered lipid membrane, while may fail to be endocytosed into normal host cells due to receptor-ligand recognition inadequacy by self-proteins capped particles.

In blood circulation, other synthetic polymer capped nanoparticles are likely to confront first with negatively charged plenty of oxidant-full erythrocytes and vascular endothelial cells. This may cause endocytosis after non-specific or blood-group antigen specific attachment to erythrocytes, resulting dose-dependent haemolysis or micro-agglutination followed by erythro-phagocytosis. Thus, excess ROS liberated into circulation, instead of intracellular ROS mediated cytotoxicity to target microbes, may cause toxicity to different host tissues after dissemination through circulation. By using blood group compatible serum as capping agent such risk can be averted. Their potent, antimicrobial activities irrespective of replicative stage and nonspecific synergism with conventional antimicrobials for higher inflow through damaged cell wall, may allow single subtoxic smaller intravenous dose for controlling intractable blood-stream infections. Based on in-vitro and in-vivo cytotoxicity studies, a predicted rational, safe antimicrobial dose can be evaluated for future trial on infected animal model or human volunteers.

## Results

### Characterization of test and control AgNPs

**Figure 1:**
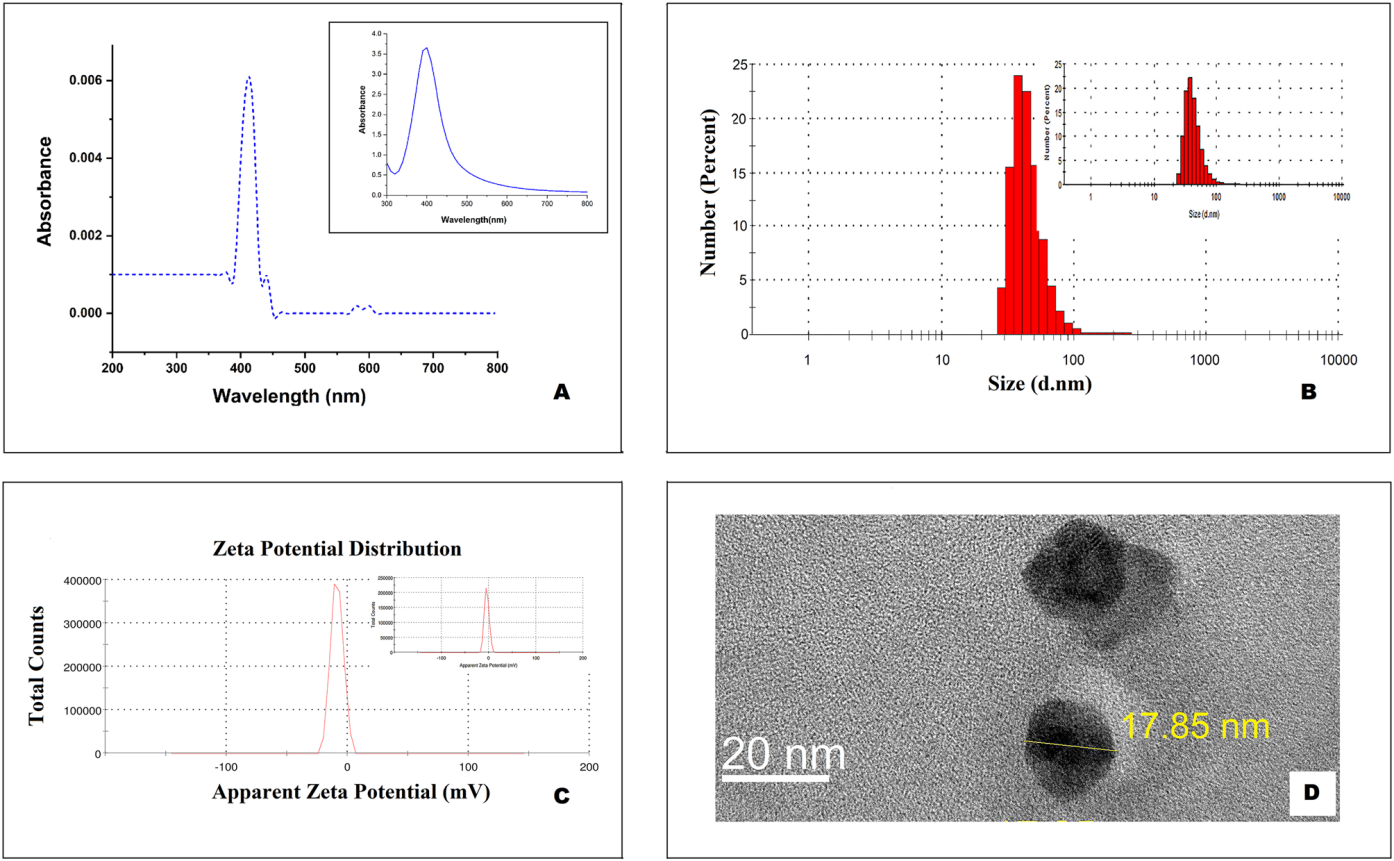
Physical characterization of AgNP-Serum-18. (**A**) UV–Vis absorption spectra of AgNP-Serum-18 from 200–800 nm; inset: high concentration (tenfold). (**B**) Cluster size obtained from DLS measurements of freshly prepared samples; inset: after 2 month. (**C**) Zeta potential of AgNP-Serum-18 of freshly prepared samples; inset: after 2 month. (**D**) TEM image.

The maximum absorption peak of AgNP-Serum-18 displayed by UV–Vis absorption spectra was around 410 nm and peak width 20 nm (Fig. 1). UV–Vis Spectra shows the absorbance for Fig. 1A (main) low concentration and for Fig. 1A (inset) tenfold high concentration of AgNP-Serum-18. Their cluster size distribution obtained from DLS (Fig. 1B) shows the average hydrodynamic diameter to be ~ 24 nm. Their cluster size distribution obtained from DLS along with negative zeta potential values − 8.9 mV. This value may be less than the accepted value of usually obtained for stable dispersion, however as our long-term measurement shows, this value remains almost unchanged over 2 months, and the same is true for the cluster size too (inset Fig. 1B,C). This shows that the dispersion has the required stability for the period of observation. Also, the fact is that the cluster size remains the same provides and indirect proof that ionic silver is not being released significantly over this period. AgNPs were almost round in shape and average size was 18 nm as demonstrated by transmission electron microscopy (TEM) images, consistent with hydrodynamic diameter. Stability of AgNP-Serum-18 was performed both by Zeta potential and consistent antimicrobial efficacy after several months of preservation.

**Figure 2:**
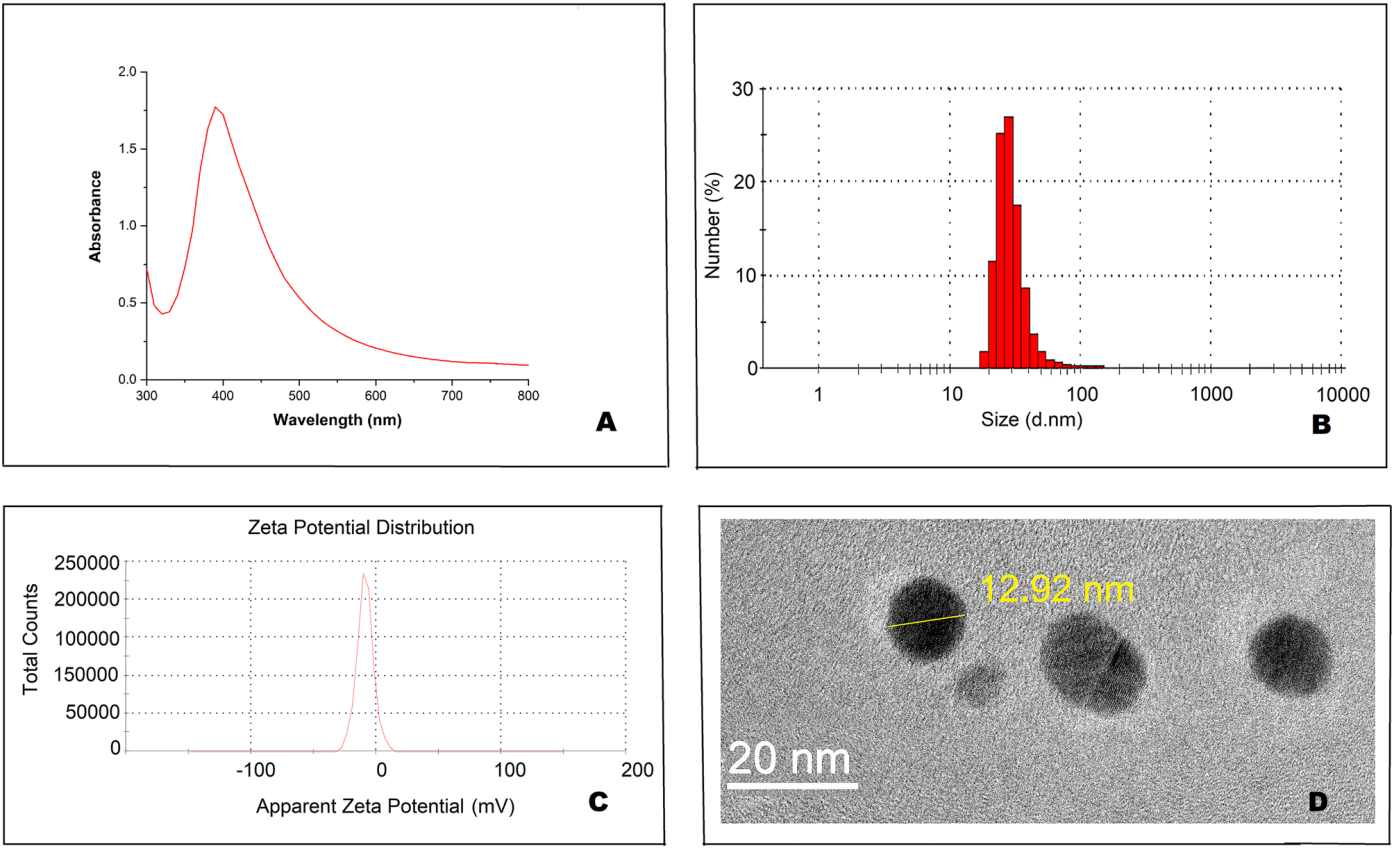
Physical characterization of AgNP-PVA-13. (**A**) UV–Vis absorption spectra of AgNP-PVA-13. (**B**) Cluster size obtained from DLS measurements. (**C**) Zeta potential of AgNP-PVA-13. (**D**) TEM image.

The maximum absorption peak of control AgNP-PVA-13displayed by UV–Vis absorption spectra was around 420 nm (Fig. 2). Their cluster size distribution obtained from DLS along with negative zeta potential values − 8.8 mV ensured high aggregation stability of such mixture of AgNPs in aqueous dispersion. AgNPs were almost round in shape and average size was 13 nm (2.D) as demonstrated by transmission electron microscopy (TEM) images. This synthetic AgNP-PVA-13 with very similar physical characteristics, was taken as “positive control” for comparing antimicrobial and other bio-functional activities^9^, after optimization of capping and reducing component.

### Antimicrobial assay

**Figure 3:**
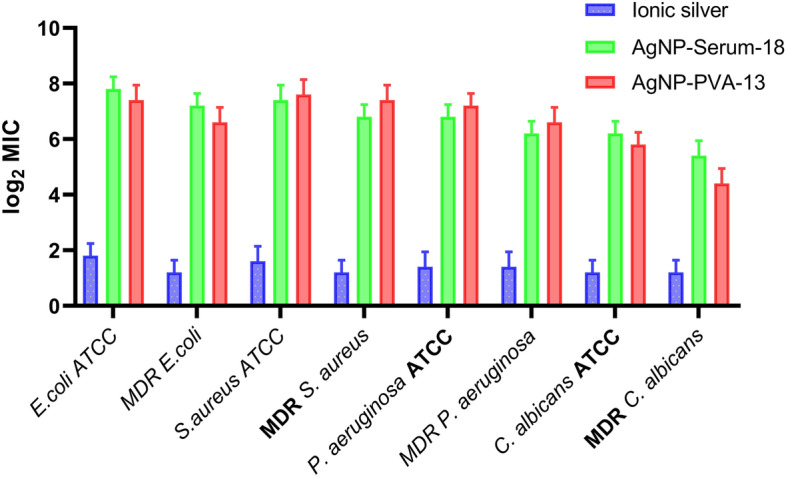
Added anti-microbial action of silver by shifting MIC (log2 MIC dilution factors 2, 4, 8, …256, 512-fold) of AgNPs and AgNO_3_ containing 107 μg silver/mL in undiluted form taken as 0 for Y axis and test organisms at X axis). Data are expressed as means ± SD. There was significant difference between the AgNP-Serum18 and Ionic silver groups (P < 0.0001).

The comparative MIC values in terms of silver contents showed 256, 256, 256 and 128 folds higher antimicrobial action respectively of test AgNP-Serum-18 (MIC between 0.02 μg and 0.04 silver/mL at 512-to-256-fold dilution), than silver nitrate solution (MIC range 5.34–2.67 μg silver/mL) (Fig. 3: bar-diagram of respective log2 dilution factors at MICs) for each of tested *E. coli*,* S. aureus*,* P. aeruginosa *and* C. albicans.* The term “bonus effect” was applied here as the “biological marker” of the nano-antimicrobial, mainly due to added antimicrobial action of Ag^0^ by creating intra-cellular oxidative stress. Thus, for all practical purpose inhibition of any one of these reference strain bacteria at concentration < 0.2 μg/mL silver (> 10 MIC for AgNPs or < 1/10th MIC of ionic silver) can be taken as nano conversion, without confirmation by complicated physical parameters. MIC values both for the sensitive reference strains and MDR strains of any bacterium were almost equal within range of biological errors for particular NPs, which indicated genetically acquired drug resistance for evading a target intervention of microbial replication, did not come on the way of nano-antimicrobial cytotoxic action. The permeation and cytotoxicity were probably related to respective cell wall integrity. Non-paired t-Test was done for the analysis of MIC of Ag-NPs against *E. coli, S. aureus, P. aeruginosa* and *C. albicans.* Significance of all the statistical tests were predetermined at P < 0.0001 using Graph Pad Prism version 8.0.2 software.

### Preservation of AgNPs

MIC values against test reference strain bacteria were noted 2 folds higher after 6 months at 4 °C refrigeration storage, whereas remained unchanged even at 6 months when preserved at room temperature in an oxygen-depleted environment. Inert gas filled sealed amber coloured container can be used for the purpose without the need of oxygen-depleted container for preservation. This indicated a fair degree of stability of such AgNP-Serum-18, at least for 3 months.

### MTT assay on cell line representing normal human liver cell line (WRL 68)

**Figure 4:**
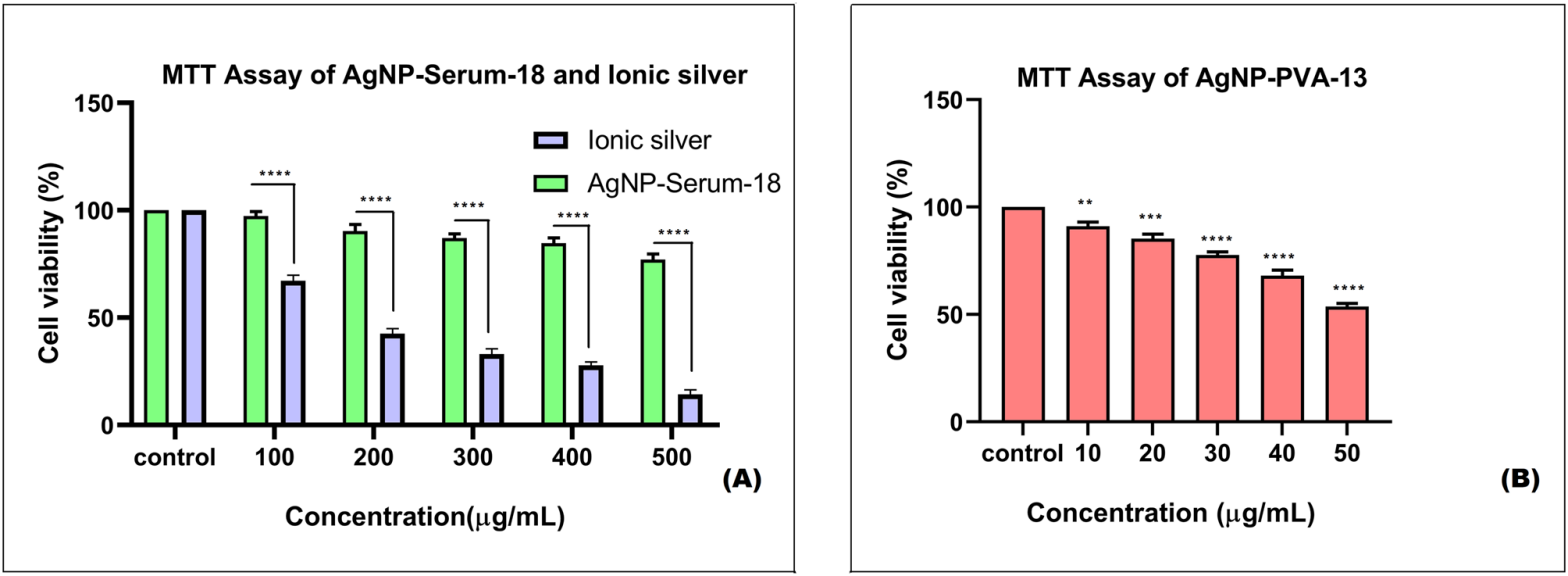
MTT Assay. (**A**) Normal liver cell line (WRL-68) with AgNP-Serum-18 and with ionic silver at equivalent concentrations as a control. (**B**) Normal liver cell line (WRL-68) with AgNP-PVA-13. Data are expressed as means ± SD. There was significant difference between the treated and control groups (**P < 0.01; ***P < 0.001; ****P < 0.0001).

MTT assay results showed almost no toxic effects of AgNP-Serum-18 on human liver cell viability even at much higher concentration (500 µg/mL) required for blood concentration (0.02–0.04 μg/mL) in antimicrobial therapy, whereas higher toxicity was noted after using AgNP-PVA-13 even at 1/10th lower concentration. MTT assay with equivalent sodium borohydride was also done, which showed remarkable toxicity. However higher toxic effects of equivalent ionic silver were obtained by MTT assay as immediate effects of excess metallic silver enter into cells by diffusion and interacted on biomolecules. On the contrary ROS mediated long-term chain reactions of programmed cell death caused by nanoparticles were not properly reflected in MTT assay. Preposition of insoluble silver into cells, derived from ionic silver may be more toxic than silver protein complex form, which are eliminated from circulation through bile and urine. Figure 4A showed there was significant difference between the AgNP-Serum-18 and Ionic silver groups (****P < 0.0001) and Fig. 4B showed there was significant difference between the AgNP-PVA-13 and control group (**P < 0.01; ***P < 0.001; ****P < 0.0001).

### Colony forming unit (CFU) assay on WRL 68 cell line

**Figure 5:**
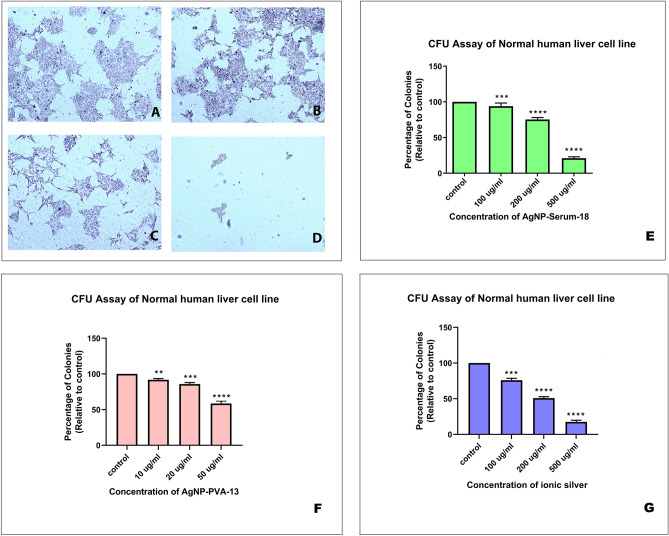
CFU assay. (**A**) Image of a normal human liver cell line (control); (**B**) cell line with the treatment of AgNPs-Serum-18 at 100 µg/mL silver equivalent concentration; (**C**) cell line with the treatment of AgNPs at 200 µg/mL silver equivalent concentration; (**D**) cell line with the treatment of AgNPs at 500 µg/mL silver equivalent concentration; (**E**) percentage inhibition of colony number on normal human liver cell line after treatment of AgNP-Serum-18 at various concentrations; (**F**) percentage inhibition of colony number on normal human liver cell line after treatment of AgNP-PVA-13 at various concentrations as a control; (**G**) percentage inhibition of colony number on normal human liver cell line after treatment of ionic silver at equivalent concentrations of AgNP-Serum-18 as a control. Data are expressed as means ± SD. There was significant difference between the treated and control groups (**P < 0.01; ***P < 0.001; ****P < 0.0001).

The extent of inhibition in terms of CFU reduction after treatment with different concentrations of AgNP-Serum-18 compared with control ionic silver solution demonstrated almost no inhibition at a concentration of AgNPs as high as 200 mg/L silver equivalent, and 50th percentile inhibition at < 500 µg/mL silver equivalent concentrations (Fig. 5). Though by MTT assay at same highest concentration showed almost no cell death, might be due to suppression of cell replication to many cells on shorter exposure or just suppression of replication by cell-signalling intervention as early event. Same inhibition study was performed for AgNP-PVA-13, where 50th percentile inhibition at > 10 mg/L silver equivalent concentrations indicating at least 50 folds higher toxicity. Figure 5E showed there was significant difference between the AgNP-Serum-18 and control groups (***P < 0.001; ****P < 0.0001); Fig. 5F showed there was significant difference between the AgNP-PVA-13 and control group (**P < 0.01; ***P < 0.001; ****P < 0.0001) and Fig. 5G showed there was significant difference between the ionic silver and control group groups (***P < 0.001; ****P < 0.0001).

### Cytotoxicity study in mice model

**Figure 6:**
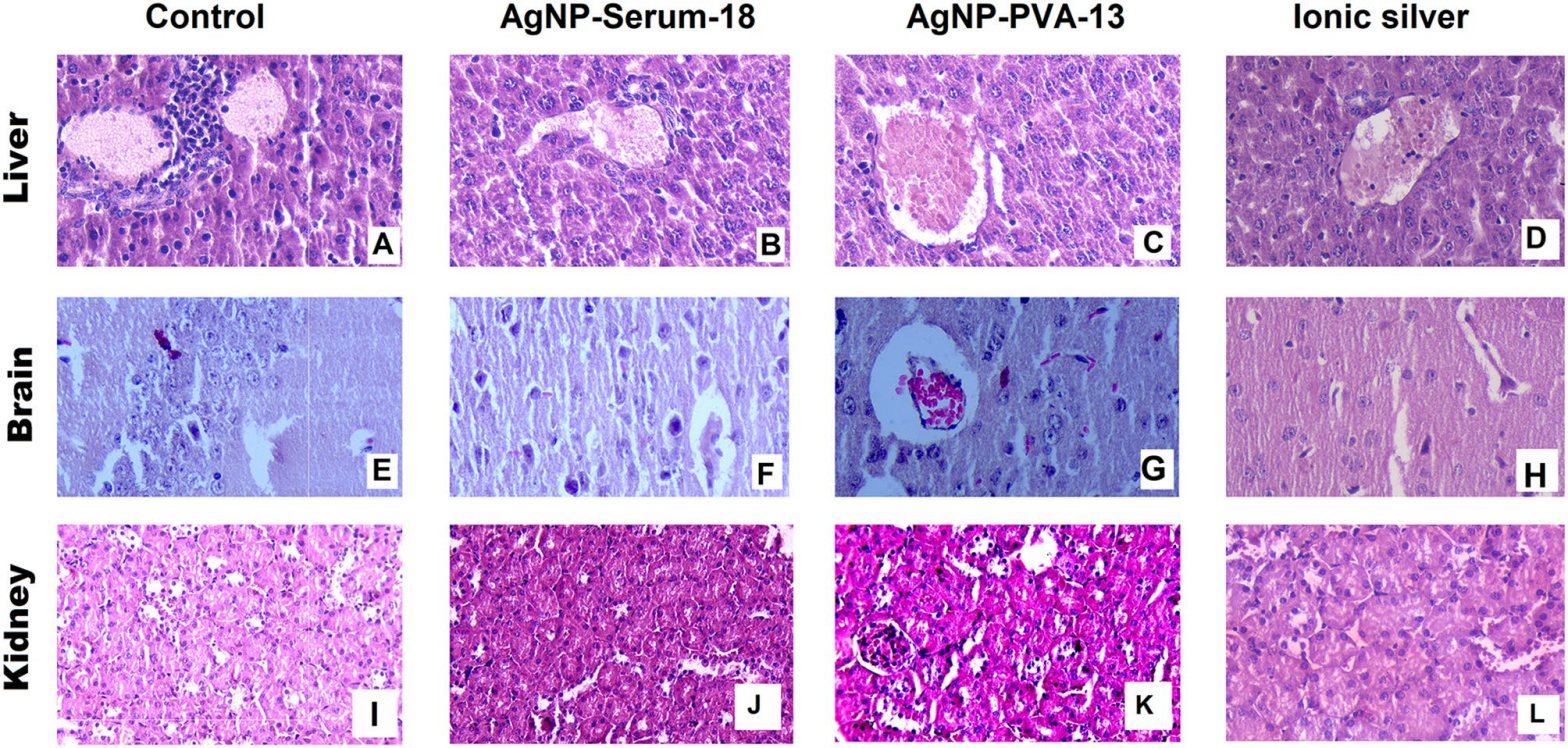
Hematoxylin and eosin (H&E)-stained liver, brain and kidney section from mice in different groups. (**A**) Liver section of mice in the control group showing normal histology and hepatocytes arranged in the hepatic cords. (**B**) Liver sections of mice in the group that received 3570 µg/kg AgNP-Serum-18 (I.V), showing: normal histology. (**C**) Liver of mice in the group that received 357 µg/kg AgNP-PVA-13 showing hepatocellular vacuolation and focal area of haemorrhage in the hepatic parenchyma. (**D**) Liver sections of mice in the group that received 3570 µg/kg ionic silver as a control (IV), showing: normal histology. (**E**) Brain section of mice in the control group showing normal histology. (**F**) Brain sections of mice in the group that received 3570 µg/kg AgNP-Serum-18 (I.V), showing: normal histology. (**G**) Brain of mice in the group that received 357 µg/kg AgNP-PVA-13 showing signs of early neuronal injury, focal inflammatory cellular infiltration and vascular congestion. (**H**) Brain sections of mice in the group that received 3570 µg/kg ionic silver as a control (I.V), showing: normal histology. (**I**) Kidney section of mice in the control group showing normal glomerulus. (**J**) Kidney section of mice in the AgNP-Serum-18 group showing no necrosis. (**K**) Kidney section of mice in the AgNP-PVA-13 group showing necrosis found in kidney tubules. (**L**) Kidney section of mice in the ionic silver as a control showing no necrosis.

No behavioural change of tested mice nor histopathological changes in liver, kidney and brain tissue (Fig. 6) were observed till sacrifice on 28th day in a preliminary study for cytotoxicity on mice model in 3570 µg silver equivalent test AgNP-Serum-18/kg body weight I.V. A similar observation was reported using bovine serum capped nanoparticles in the rat model^10^. It also reiterates that human AgNP-Serum-18 at low dose is well-tolerated in the murine model. For technical reason, the test could not be carried out with mouse AgNP-Serum-18 and at higher dose of nano preparations to obtain end point toxicity evaluation. The cytotoxic effects of AgNP-PVA-13 were pronounced and all tested mice at 35.7 µg/kg test dose (1/100th.) died within 1st week with focal haemorrhage and necrosis in different organs (Fig. 6). Results indicated appreciable safety limit (> 100) of AgNP-Serum-18. similar toxic effects were noted with most other AgNP-PVA-13^11^, though studies showed gradual diminution of their bio-functional properties in blood circulation by acquiring corona protein coat.

### Effect on blood biochemistry

AgNP-Serum-18 and AgNP-PVA-13 [1/10th] were injected into blood stream of two different sets of mice. From three mice of each set, orbital-sinus blood samples were collected by capillary tubes under local anaesthesia for blood biochemistry evaluation on 3rd day, along with control groups. AgNP-PVA-13 showed significantly higher toxic effects on functions of different organs even at 1/10th concentration, when compared to the control group and AgNP-Serum-18 (Table 1).

**Table 1:** Serum biochemical values of Swiss albino male mice after 4 weeks of AgNPs administration (n = 4). ^1^blood urea nitrogen; ^2^creatinine; ^3^total bilirubin;^4^conjugated bilirubin; ^5^unconjugated bilirubin; ^6^alkaline phosphatase; ^7^aspartate amino transaminase; ^8^alanine amino transaminase.

Dose (µg/kg)	Control	Silver 3570 (AgNP-Serum-18)	Silver 357 (AgNP-PVA-13)
BUN^1^ (mg/dL)	109.75 ± 7.5	139 ± 12	280 ± 17
CRE^2^ (mg/dL)	0.45 ± 0.15	0.5 ± 0.1	1.675 ± 0.2
TBIL^3^ (mg/dL)	0.225 ± 0.15	0.2 ± 0.1	1.025 ± 0.35
CBIL^4^ (mg/dL)	0.02 ± 0.015	0.0325 ± 0.1	0.37 ± 0.36
UNCBIL^5^ (mg/dL)	0.015 ± 0.005	0.035 ± 0.005	0.08 ± 0.01
ALP^6^ (IU/L)	144.75 ± 14	148.5 ± 13.5	255.5 ± 26
AST^7^ (IU/L)	164.75 ± 44	144.25 ± 16.5	378 ± 12.5
ALT^8^ (IU/L)	63 ± 3.5	62.75 ± 6.5	73.5 ± 12

All data were presented as the mean ± SEM. Data were subjected to statistical analysis using one-way ANOVA with GraphPad Prism 8.0.2(263). Mean values were considered to be statistically significant at *P* < 0.05.

## Discussion

By in-vitro studies many heavy metal nanoparticles have been proved as wide range, potent antimicrobial agents^12^, of which AgNPs have been extensively studied and believed as more stable, less toxic, broad-spectrum, apparently resistant-proof potent antimicrobial agents. Our prepared AgNP-Serum-18 has also shown potent anti-microbial action against tested common pathogenic bacteria and yeast. During wet chemical preparation, the reducing agent gives electrons to silver ions (Ag^+^) to form unstable silver atoms (Ag^0^), which tend to form unstable clusters of uncapped silver nanoparticles. By electrostatic stabilization, surfactant polymers prevent the uncontrolled amount of particle formation and particle aggregation by steric repulsion, giving rise to nano-sized (1–100 nm) colloidal particles containing a core of aggregated silver atoms capped by stabilizing molecule with adsorption of ions to the surface. This creates an electrical double layer that impairs Columbic repulsion force between individual particles. That electrostatic force is expressed as Zeta potential of colloidal nanoparticles and is an important guiding force to interact with other cells with great differences of electrostatic surface charges. AgNP-Serum-18 has been prepared with mild reducing agent dextrose and serum as mixture of many capping and reducing agents, eventually resulted all physical and bio-functional properties of one good anti-microbial nanoparticles. Added advantage of this is that, surplus reducing and capping agents need not be removed before in-vivo studies as same dextrose and serum are natural components of blood.

Such nanoparticles can bind to the bacterial surface mainly by electrostatic forces^13^ and split bi-layered lipid membrane with a special affinity to negatively charged lipopolysaccharide of Gram-negative bacteria^14,15^. This, in turn, causes greater permeability, osmotic disbalance and release of cytoplasmic contents. Silver atoms attack the respiratory chains at mesosome level of the bacterial cell membrane, resulting in cell death. Such antibacterial activities are mainly mediated by reactive oxygen species (ROS) and found to be increased in the presence of oxygen and decreased in the presence of antioxidants^16^. Similar oxidative stress by excess production of ROS after interaction with Ag^0^ can happen inside the mammalian cell at mitochondria level, through the reaction of silver with the thiol group of the enzymes^17^. Also, ROS can cause dephosphorylation of peptides on tyrosine residues and promotes cell death by influencing bacterial signal transduction^7,18^. Such ROS mediated “programmed cell death” may require longer time to complete chain reactions, compared to direct cytotoxic action of heavy metals. However, internalization of nanoparticles into mammalian cells predominantly occurs by endocytosis through receptor-ligand interactions, while ionic heavy metals enter by gradient dependent diffusion. So, toxicity of nanoparticles to mammalian cells is primarily regulated by structure of surface capping molecules^19,20^. Due to large surface area to volume ratios, large Ag^0^ payloads can be delivered by small size triangular silver nanoparticles than larger one^21^. Their size and shape depend upon the method of nano preparation and used stabilizing molecules for the purpose. For all these reasons AgNP-Serum-18 has shown strong antimicrobial action but appreciably low toxic action on mammalian cells.

The minor protein compositions of human serum differ from one individual to others in terms of blood group iso-antibodies, acquired immune-bodies following vaccination or infection and auto-antibody in persons with auto-immune disorders. Their inclusion as capping agents in AgNP-Serum-18may have adverse effects by targeting selective host cells, if mismatched. This may explain dose-dependent haemolysis by polyvinyl pyrrolidone and citrate capped AgNPs^22^ with the observation of ROS mediated membrane lipid damage^23^, leading to haemolysis^24^. If such nanoparticles target non nucleated human RBCs^25^ and release sufficient free Ag^0^ or ROS into circulation before attachment with target microbes, may also cause many unpredictable adverse effects to other cells of the host. For this reason, we recommend the use of matched serum preferably with antibody of specific infection. Disease-specific monoclonal antibodies can be added to make these more target specific microbicidal agents. Similarly, vascular endothelial cells with strong negative surface charge can be first target of positive-charged nanoparticles. As vascular wall is covered with endothelial glycocalyx supplemented with components of plasma, may restrict entry of plasma/serum capped nanoparticles. Thus, it may open-up the immense possibilities of therapeutic applications by a new class of nano-composites. Earlier workers also emphasized the role of surface capping agents of noble metal nanoparticles for molecular recognition of target cells^26^. A nano-silver probe for immunoassay has also been developed by Yeh et-al.^27^ by using IgG antibody in citrated capped AgNPs, So, disease-specific antibodies on the surface of systemic usable AgNPs should be recommended for selective destruction of target cells or microbes. With all these preambles, test AgNP-Serum-18 can be a promising non-specific microbicidal nano-medicine, safe for intravenous use. Results of cytotoxicity studies also support this.

Other factors for prohibiting systemic use of many non-serum-capped nanoparticles were, instability or short half-life, unmatched viscous colloidal state of the preparation and adsorption of various essential protein components from circulation^28,29^ In the experiment with bovine albumin capped AgNPs, corona formation with protein molecules at their surface^30,31^ was observed and such AgNPs caused least haemolysis than others^32,33^. Huang H et-al.^22^ also demonstrated 20 different corona proteins after incubating citrate and PVP capped AgNPs with plasma, including immune-globulin fragments. So, self-serum or plasma was chosen as most suitable capping agent for systemic use not only to guard haemolysis but also for the selective attack to microbes against which person carries antibodies. Nanoparticles prepared from blood group matched serum of convalescence patients may also be used as life-saving infection controlling agent. During intravenous use of nanoparticles, nucleus and mitochondria free human RBCs are first target, being a bag of oxygen carrying molecules. So, AgNP induced ROS coming into circulation even after depletion of 0.1% cells, can cause sufficient toxic effects to the host by endothelial damage, platelet aggregation, phagocytic oxidative burst and genotoxicity. This may be the major reason of toxicity for earlier nano preparations, though these become gradually less hemolytic after acquiring plasma derived corona coats. For same reasons, the test AgNP-Serum-18 is proved much safer than equivalent silver containing AgNP-PVA-13, by our various test parameters on mice and human liver cell line.

After human trials, therapy with appropriate safe serum coat nanoparticles can be introduced as life-saving measures for MDR bacterial or fungal infections (including MDR *Candida auris*, Mucor-mycosis) and some viral infections, like hepatitis B, AIDS, Ebola, COVID-19 etc. Even rabes patients may be treated without reversion of neural damages, by suitable nanoparticles capped with a mixture of self-serum and hyper-immune serum. Such drug may be ready answer for new emerging pathogens in days to come.

For instant preparation of such self-serum-based nano-antimicrobials, an automated vending machine may be designed where ingredient solutions can be mixed sequentially at required physical conditions.

Plasma or whole blood collected with dextrose-citrate solution may be similarly tested for nano-conversion. However, serum is preferred because nano-size and bio-functional efficacy may be compromised with further incorporation of coagulation factors on capping materials. Other potent heavy metal nano-antimicrobial particles may also be made therapeutically useful after treating with self-plasma/serum in starring condition to convert into “secondary novel nanoparticles”. However, they may be toxic at uncapped state.

In earlier studies, different AgNPs showed nonspecific synergism^34–36^ with conventional antibiotics and potent anti-biofilm properties^37^. This can be advantageously used in therapeutic planning of combination therapy for low dose use of both drugs. Alteration of cell membrane permeability in damaged nano treated microbial cells may be the primary reason for non-specific synergism for other drugs. As the mechanism of antimicrobial action of nanoparticles is independent of cell replication, these can also kill dormant, non-replicating microbes within biofilms. This may be an important reason for antibiofilm role and action on non-replicating cells like human RBC.

A proposed single-dose infusion of 100 mL AgNP-Serum-18 may be theoretically enough^38^ for managing intractable infections in terms of higher MIC achievable concentration in blood for most in-vitro tested MDR strains, keeping the wide margin of safety for the host. Interpolating results of in-vitro and in-vivo toxicity study, the same is indicated. The amount of silver delivered with 100 mL AgNP-Serum-18 is only 10.8 mg (approximate achievable plasma concentration > 2 mg/L sufficient for antimicrobial action, while 0.1 mg silver/kg body weight is well tolerable by the host). This, after oxidation within cells, are likely to be eliminated from body through kidney as ionic silver and may not be harmful for environment. Even by using 1/10th of AgNPs (10 mL), synergistically with one apparently resistant antibiotic, desired infection control may be possible. This is supported by an in-vitro study adding 1/4th MIC carboxy-methyl cellulose capped AgNPs at ultra-dilution in emulsion fluid of automated susceptibility testing device that reverted all resistant drugs into sensitive range^34^. For preparation of this 10 mL AgNPs, only 2.5 mL patient’s serum will be required, which can be easily collected from any adult patient.

Many workers prepared nanoparticles by “green bio-synthesis” using various plant or microbial extracts as stabilizing agents and claimed as safer. But such organic coating agents were only suitable for topical use or surface disinfectants or pesticides, but not suitable for systemic application^39^. However, their excessive spillage into soil or water-bodies may create long-term adverse effects on ecosystem. Our AgNP-Serum-18 can also be considered as one type of green bio-synthetic product, but safer for systemic use. Many workers prepared AgNPs using trehalose^40^ and other reducing^41^ or capping agents^42–44^ mainly suitable for developing biosensors to apply in biomedical or environmental science, but may not be suitable for therapeutic use.

## Methods

### Chemicals and biological materials

For the synthesis of two different AgNPs, silver nitrate (99.9%, Sigma Aldrich, USA) was taken along with dextrose (Sigma Aldrich, USA) and sodium borohydride (Sigma Aldrich, USA) as reducing agent. As capping agent, pooled, “O” Rh-D positive blood group human serums from discarded samples of healthy individuals were taken as capping agents, after sterilization by passing through the membrane filter. Poly vinyl alcohol (PVA, MW: 60,000–1,50,000) (Hi-media, India) was used as a primary capping or anionic surfactant stabilizing agent for preparation of another AgNPs.

For the determination of respective MICs of AgNP-Serum-18 against different microorganisms, their Mueller–Hinton broth cultures (Hi-media, India) were used. The following standard reference strains (Microbiologics-Inc, USA) were used: *Escherichia coli* ATCC 25922, *Pseudomonas aeruginosa* ATCC 27853 and *Staphylococcus aureus ATCC 25923 Candida albicans* ATCC 10231. Clinical isolates of respective multidrug resistant microbes from blood samples of sepsis patients were included after isolation, identification and antimicrobial susceptibility testing in the automated system in our laboratory.

For Cell culture, HeLa derivative Normal human liver cell line (WRL-68) similar to human hepatocyte cell were used procured from NCCS (Pune, India) was taken for cytotoxicity study. Cell line was cultivated in Modified Eagle’s medium (MEM) (11095080, Gibco) supplemented with the 10% FBS (16140063, Gibco) and 1% antibiotics (penicillin/100 U/mL and streptomycin/100 μg/mL).

### Preparation of systemic usable AgNPs

Wet chemical reduction method^45^ was followed to prepare systemic usable AgNP-Serum18. Pooled, “O” Rh-D positive blood group human serums was used as a primary capping or anionic surfactant stabilizing agent and dextrose as a reducing agent. Actually, serum was used as a mixture of various capping protein molecules and some reducing substances. So, the presence of such cock-tail stabilizing molecules in excess only ensured excess stabilization of the product without increasing toxicity. Discarded human serum sample after testing in serology department were collected for nano preparation with consent from head of the Institution. All methods were carried out in accordance with relevant guidelines and regulations. All experimental protocols were approved by Institute of Post Graduate Medical Education & Research (IPGME&R), Kolkata, India.

The aqueous solution of silver nitrate (AgNO_3_) was prepared in deionized water at 4 mM concentration (672 μg/mL AgNO_3_) so that effective concentration of silver in nano preparation resulted to 1 mM (106.8 μg/mL silver or 168 μg AgNO_3_/mL) concentration. Five mL of serum (pH 7.4) was taken in 50 mL beaker. This serum stirred and heated at 56℃ to obtain decomplemented serum solution. Then 5 mL of 4 mM AgNO_3_ solution was added to the serum solution under continuous stirring to obtain Ag^+^/serum complex solution. 10 mL of 2 mM dextrose solution was added to the Ag^+^/serum complex solution under continuous stirring. The temperature of solution increased to 75 ℃. The optimal nano-transformation was indicated by the change; colour to light brown. To avoid light and oxygen exposure as far as practicable, the resultant mixture was stored^46^ in the suitably small air-tight amber-coloured bottle without much air space. Product was preserved at 4 °C refrigerator before use within 3 months.

Same synthesis procedure was followed for animal study and cell line study, where 40 mM AgNO_3_ and 20 mM of dextrose was used as reducing agent. Where 5 mL of 40 mM AgNO_3_ solution was added into 5 mL of serum solution. After stirring the Ag^+^/serum complex solution, 10 mL of 20 mM dextrose solution was added to the complex solution under continuous stirring. Their mixing ratio was 1:1:2 (v/v) so that effective concentration of 10 mM silver equivalent nano could be obtain for a higher dose toxicity evaluation study in smaller animal. Physical characterization by DLS showed no remarkable difference in particle size and MIC values against reference strain bacteria also remained same. Removal of excess reducing or capping agents were not required for injecting the product into blood where same components remained plentiful.

### Preparation of PVA capped AgNPs as positive control for toxicity study

AgNP-PVA-13were synthesized by reducing silver nitrate with sodium borohydride and adding polyvinyl alcohol as the capping agent to control the growth of nanocrystals and agglomeration of nanoparticles. Aqueous solutions containing 0.24% (by weight) PVA was heated to 100ºC to obtain homogeneity and cooled down to room temperature before use. With 15 mL PVA solution 5 mL of 4 mM AgNO_3_ was added to obtain 1 mM solution of silver salt. AgNP-PVA-13 were prepared by rapidly injecting 0.3 mL of 10 mM NaBH_4_ into 20 mL PVA solution containing 4 mM AgNO_3_ at room temperature. After 10 min of stirring, the reaction mixture was stored at 4 °C before use. Any toxicity contributed by excess of reducing substance was checked separately with equivalent concentration of sodium borohydride.

### Characterization of AgNPs

The hydrodynamic size of prepared AgNP-Serum-18 was determined by UV–Vis absorption spectrophotometer (Thermo Scientific Genesys 10S Vis). By lognormal size distribution curves obtained from DLS (Malvern Zen 3600 Zetasizer, USA) measurements, the size ranges of nano mixture with narrow size distributions were indicated. High aggregation stability of such mixture of AgNPs in aqueous dispersion was indicated by suitable negative zeta potential (Malvern Zen 3600 Zetasizer, USA) values. However, the actual size and shape were determined by transmission electron microscope (JEOL JEM 2100 HR with EELS, USA) images.

The physical characterization of AgNP-PVA-13 was similarly performed.

### Silver nanoparticles formulation

Silver nanoparticles of an average particle size of 18 nm was taken from our synthesized AgNP-Serum-18, in a solution form containing about 100 µg silver/mL. The stock solution was first tenfold diluted, then followed two-fold serial dilutions for antimicrobial efficacy study at eight different adjusted two-folds diluted concentrations of 10, 5, 2.5, 1.25, 0.625, 0.312, 0.156, 0.078 µg silver/mL.

From molecular weight of any salt, the molarity in aqueous solution is calculated. In silver nitrate of MW ~ 170 single atom of silver represents ~ 107, thus silver equivalent in 0.1 mM solution can be calculated as ~ 10.7 mg/L. Calculated molecular weight of heavy metal in respective salt. For heavy metal nanoparticles different heavy metal salts or different salts of same heavy metal are used. For comparison of bio-functional efficacy, equivalent heavy-metal content in solution can be better indicator than whole salt. Similarly equivalent ionic silver solution containing same amount of silver can be prepared adjusting salt and diluent ratio.

### Antimicrobial activity and determination of MIC of AgNPs

Extensive antimicrobial studies were conducted for AgNP-Serum-18 against test organisms following standard broth dilution method (CLSI M07-A10)^41^. The MIC was determined in MH broth using serial two-fold dilutions of Ag-NPs in concentrations ranging from 0.078 to 10 μg/mL (silver equivalent) with adjusted bacterial concentration at 0.5 McFarland’s standard (0.10 OD at 625 nm) equivalent to about 1 × 10^8^ CFU/mL. The positive control used in this study bacteria inoculated MH broth medium and negative control uninoculated MH broth. The time and temperature of incubation was 24 h and 37 °C respectively. The MIC is the lowest concentration of antimicrobial agents that visually inhibits 99% growth of microorganisms. The MIC was noted by the visual turbidity of the tubes both before and after incubation and it was done in five sets to confirm its value for the tested bacteria.

The end-point inhibition levels in two rows containing nano silver solution and equivalent 1/10th. diluted ionic silver solution was noted. Higher antimicrobial actions of AgNPs over equivalent ionic silver were taken as “Bonus Effects” for different bacteria/yeast; those indicated additional microbicidal mechanisms^33^.

### Statistical analysis

The data thus obtained were analyzed by using descriptive statistics including mean and standard deviation. Non-paired t-Test and one-way ANOVA was done for the analysis. Significance of all the statistical tests were predetermined at P < 0.05 using Graph Pad Prism version 8.0.2 (263) software.

### Effects on conditions for the preservation of nano-antimicrobials

Antimicrobial action of prepared AgNPs was determined at monthly intervals up to 6 months by observing any change of MIC values for fixed test organism (*Escherichia coli* ATCC-25922) at 1/4 MIC to 4 MIC range micro-broth dilution study. Stock AgNPs were preserved aerobically at room temperature and refrigeration as well as in hydrogen free oxygen-depleted chamber^2,47^ Simultaneous five repetitions of the study were done to validate results.

### MTT assay on normal liver cell line (WRL 68)

Cell viability was measured by the MTT colorimetric technique with minor adjustment^3,48^. In brief, in each well, 200 μL of MTT [3-(4,5-dimetheylthiazol-2)-2,5 diphenyl tetrazolium bromide] without phenol red, yellowish in colour solution (5 mg/mL in PBS) was applied to each well containing normal liver cell line in MEM medium with or without varying effective concentrations of AgNP-Serum-18 (test) or AgNP-PVA-13 (positive control) and proportionate silver-nitrate with sodium borohydride (negative control) in separate sets. Plates were incubated for 6–7 h in a 5% CO_2_ incubator for the reduction of MTT by metabolic active cells with formation of purple formazan.

Isopropanol or DMSO was added100 μLto each well for the solubilization of MTT crystals. The plates were placed on a shaker for 15 min for complete crystal solubilization and the optical density of each well was then calculated. The quantity of the formazan product was measured by 595 nm using the scanning Multi-well spectrophotometer (Biorad, Model 680, Japan) with DMSO as blank. Absorption OD values proportionately indicated the number of living cells in culture.

The relative cell viability (percent) of the control well containing the cell culture medium without nanoparticles as a vehicle was determined A-test/A-control × 100; where A-test is the absorbance of the test sample and A-control is the absorbance of the control sample. Each measurement was repeated three times independently in quintuple to calculate the standard error. Statistical analysis was performed using the Student’s *t* test.

### CFU assay

HeLa derivative Normal human liver cell line (WRL-68) were used and procured from NCCS (Pune, India) was taken for cytotoxicity study. Two mL of MEM medium was added into each well (flat bottom 6 wells tissue culture plate), followed by addition of 10 µL of confluent cells. After 24 h incubation at 37 °C in CO_2_ incubator, plates were checked for cell adherence which was continued for another 3 days incubation to observe colony formation. After confirming colony formation, wells were washed with incomplete MEM medium (without FBS). Then, up to 4 mL of fresh complete MEM medium were added with the addition of tenfold strong AgNP-serum-18, 0 mL (control), 0.37 mL, 0.75 mL and 1.85 respectively. Thus, approximate effective concentrations of silver were achieved to 0, 100, 200 and 500 µg/mL at wells in chronological order. Again, after 24 h incubation at 37 °C in CO_2_ incubator, wells were washed with PBS, then fixed with 500 µL of methanol for 15 min. Wells were washed twice with PBS, then stained with Hematoxylin for 2 min, followed by washing in tap water. Cell line imaging was done at 4× inverted microscope. Tests were repeated times independently and CFU % inhibitions in respect of control were calculated. Statistical analysis was performed using the Student’s *t* test.

### The margin of safety study in the animal model

Fifteen male Swiss albino mice of 4–5 months of age with 25–30 g body weight were taken, five for every three groups. Tenfold higher AgNPs were prepared for small animal experiment and checked for comparable antimicrobial action at 1/10th dilution. First test group mice were treated with 100 µL of tenfold-strong AgNP-Serum-18 (an attempt to reach a highest possible toxic dose) as single intravenous injections, with second untreated group mice as negative control. Third group was treated with 100 µL of AgNP-PVA-13 containing 1/10th silver equivalent toxic nanoparticles as positive control. By this way, about 107 µg silver equivalent test nanoparticles and 1/10th AgNP-PVA-13 (10.7 µg silver equivalent) were given per mouse by I/V route which were equivalent to 3570 µg/kg and 357 µg/kg body weight, respectively. Their behavioural changes were noted daily, and one mouse from each group of test and negative control were sacrificed on 28th day, while mouse from positive control (AgNP-PVA-13) was taken on day of death. All mice died within few days of AgNP-PVA-13 injection which showed profound toxicity in mice model, both by histopathological and blood-biochemical parameters.

Food was withheld for live mice, 24 h before necropsy. The mice were anesthetized with Ketamine 22–24 mg/kg IM route as per CPCSEA guidelines for laboratory animal facility.

For histopathological examinations of liver, kidney and brain tissues, each was aseptically dissected. From sections of formal-saline preserved tissues, Eosin-Haematoxylin-stained slides were prepared and examined microscopically to note any haemorrhage and necrotic features difference than that of control.

### Biochemical and hematological evaluation

On 3rd. day of experiment, three mice from each set receiving IV injections were taken. Blood samples were drawn in capillary tubes from orbital sinuses by ocular puncture and analyzed for BUN (blood urea nitrogen); CRE (creatinine); TBIL (total bilirubin); CBIL (conjugated bilirubin); UNBIL (unconjugated bilirubin); ALP (alkaline phosphatase); AST (aspartate amino transaminase); ALT (alanine amino transaminase) using a biochemical blood analyzer.

### Ethics statement

Discarded human serum sample after testing in serology department were collected for nano preparation with consent from head of the Institution and approved by the Institutional Ethics Committee at Institute of Post Graduate Medical Education & Research (IPGME&R), Kolkata-20, India (Ethical No. Inst/IEC/2018/488). All animal experiments and sample collection were performed at Chittaranjan National Cancer Institute (CNCI). The protocols were approved by the Institutional Animal Ethics Committee (IAEC) at CNCI (Ethical No.- IAEC-1.1/2018/AH-35, 01.09.2017-01.09.2020) and all manuscript confirming the study was carried out in compliance with the ARRIVE guidelines.
